# Mediation of Cardiac Macrophage Activity *via* Auricular Vagal Nerve Stimulation Ameliorates Cardiac Ischemia/Reperfusion Injury

**DOI:** 10.3389/fnins.2020.00906

**Published:** 2020-09-08

**Authors:** Chee Hooi Chung, Beatrice Bretherton, Satirah Zainalabidin, Susan A. Deuchars, Jim Deuchars, Mohd Kaisan Mahadi

**Affiliations:** ^1^Drug and Herbal Research Centre, Faculty of Pharmacy, Universiti Kebangsaan Malaysia, Kuala Lumpur, Malaysia; ^2^School of Biomedical Sciences, Faculty of Biological Sciences, University of Leeds, Leeds, United Kingdom; ^3^Programme of Biomedical Science, Center for Toxicology and Health Risk Study (CORE), Faculty of Health Sciences, Universiti Kebangsaan Malaysia, Kuala Lumpur, Malaysia

**Keywords:** auricular vagal nerve stimulation, macrophage polarization, myocardial infarction, cholinergic anti-inflammatory pathway, ischemia/reperfusion injury

## Abstract

**Background:**

Myocardial infarction (MI) reperfusion therapy causes paradoxical cardiac complications. Following restoration of blood flow to infarcted regions, a multitude of inflammatory cells are recruited to the site of injury for tissue repair. Continual progression of cardiac inflammatory responses does, however, lead to adverse cardiac remodeling, inevitably causing heart failure.

**Main Body:**

Increasing evidence of the cardioprotective effects of both invasive and non-invasive vagal nerve stimulation (VNS) suggests that these may be feasible methods to treat myocardial ischemia/reperfusion injury *via* anti-inflammatory regulation. The mechanisms through which auricular VNS controls inflammation are yet to be explored. In this review, we discuss the potential of autonomic nervous system modulation, particularly *via* the parasympathetic branch, in ameliorating MI. Novel insights are provided about the activation of the cholinergic anti-inflammatory pathway on cardiac macrophages. Acetylcholine binding to the α7 nicotinic acetylcholine receptor (α7nAChR) expressed on macrophages polarizes the pro-inflammatory into anti-inflammatory subtypes. Activation of the α7nAChR stimulates the signal transducer and activator of transcription 3 (STAT3) signaling pathway. This inhibits the secretion of pro-inflammatory cytokines, limiting ischemic injury in the myocardium and initiating efficient reparative mechanisms. We highlight recent developments in the controversial auricular vagal neuro-circuitry and how they may relate to activation of the cholinergic anti-inflammatory pathway.

**Conclusion:**

Emerging published data suggest that auricular VNS is an inexpensive healthcare modality, mediating the dynamic balance between pro- and anti-inflammatory responses in cardiac macrophages and ameliorating cardiac ischemia/reperfusion injury.

## Introduction

Ischemic heart diseases, particularly myocardial infarction (MI), remain a primary factor for mortality and morbidity worldwide. A recent observational study of a European cohort revealed similar mortality rates (∼49%) for both acute and chronic MI, highlighting the poor prognosis (Kadesjö et al., 2019). The American Heart Association reported that one third of total deaths for individuals over 35 years of age in the United States were associated with cardiovascular disorder (CVD) (Benjamin et al., 2018). The disease progression is alarming, as 43.5% of adult Americans are projected to have CVD in 2035 with public health expenditure potentially reaching up to USD 1.1 trillion.

Initial therapy for MI aims to promptly reperfuse the infarct myocardium. This can be achieved pharmacologically through thrombolytic therapy to break up clots within the artery or percutaneous coronary intervention to widen the narrowed artery (Rentrop and Feit, 2015). However, ischemia/reperfusion (I/R) injury is paradoxical to the restoration of the blood flow and is associated with poor prognosis in MI patients (Huang and Frangogiannis, 2018). This is partially due to augmented inflammation during post-reperfusion therapy and exaggeration of apoptosis (Yao et al., 2019). To date, few anti-inflammatory agents are used in post-MI management despite strong association of inflammatory cytokine imbalance with MI pathogenesis (Huang and Frangogiannis, 2018). Early attempts showed a lower risk of ischemic cardiovascular events in MI patients with daily administration of colchicine, a potent anti-inflammatory medication (Tardif et al., 2019). The cardiac benefits of anti-inflammatory agents remain controversial since non-steroidal anti-inflammatory drugs (NSAIDs) increase the likelihood of first admission for cardiovascular events in patients with no history of heart disease (Huang et al., 2019). There is also a tendency toward fatal infection in MI patients with multiple comorbidities following monoclonal antibody treatment targeting interleukin-1β, highlighting the need for careful clinical considerations (Ridker et al., 2018).

There is growing interest in the utilization of vagal nerve stimulation (VNS) as a non-pharmacological approach in mitigating inflammation in myocardial I/R injury (Wang et al., 2014; Uitterdijk et al., 2015; Yu et al., 2017). The interplay between the peripheral anti-inflammatory response and central nervous activation is thought to be mediated *via* a cholinergic anti-inflammatory pathway (CAP). This concept was first reviewed by Tracey (Tracey, 2002), indicating that excessive cytokine production in response to microbial invasion or injury is attenuated by VNS. At the cellular level, the neurotransmitter acetylcholine (ACh) is released following vagal activation, modulating both electrical and mechanical functions of the heart. ACh binds to the α7 nicotinic acetylcholine receptor (α7nAChR), an ionotropic receptor that presents in the endothelial layer of heart tissues to modulate tonic activity of the heart (Mazloom et al., 2013). α7nAChR also presents widely in immune cells, specifically in the myocardium. In MI-induced rats, activation of α7nAChR was linked with immunomodulation, cardioprotection, reversal in MI prognosis, including sympathetic hyperactivity as measured by heart rate variability (HRV), and improved cardiac hemodynamic function (Bezerra et al., 2017). Indeed, the autonomic balance between sympathetic and parasympathetic branches is pivotal in regulating the normal physiological functions of the cardiovascular system—elevated sympathetic and decreased parasympathetic activity is a common hallmark following MI (Yokoyama et al., 2017). Significant experimental evidence indicates that VNS provides cardioprotection against myocardial I/R injury (Wang et al., 2014; Uitterdijk et al., 2015). The cardioprotective properties attributed to direct VNS in MI animals include improvement of ventricular function such as a reduction of ventricular tachycardia and ventricular arrhythmia, reduction of infarct size, attenuation of adverse cardiac remodeling, improvement of left ventricular ejection fraction (LVEF), and attenuation of myocardial interstitial fibrosis (Uemura et al., 2010; Uitterdijk et al., 2015; Zhang et al., 2016). In humans, delivering transcutaneous VNS (tVNS) at the tragus provided equivalent cardioprotection in patients with ST elevated myocardial infarction (STEMI) treated with primary percutaneous coronary treatment (Yu et al., 2017). This therefore aids with generating an initial evidence base of the cardioprotective effects non-invasive vagal stimulation can confer in MI patients (Wang et al., 2014; Stavrakis et al., 2017; Zhou et al., 2019). While numerous studies reveal the therapeutic benefits of invasive and non-invasive VNS in I/R injured myocardium, the underlying cardioprotective mechanism remains to be elucidated. In this review, we will focus on the anti-inflammatory effects of VNS/tVNS specifically in the macrophage subpopulations and how VNS/tVNS may provide therapeutic benefit in cardiac I/R injury.

## Inflammatory Cell Infiltration as Hallmarks for Cardiac Ischemia/Reperfusion Injury

After restoring perfusion, a sterile inflammatory response is activated to clear necrotic cellular debris. This inflammatory response is characterized by endothelial barrier dysfunction and the recruitment of inflammatory cells immediately after reperfusion. The whole process is initiated by the release of inflammatory signals from an extracellular matrix known as danger-associated molecular patterns (DAMPs). The release of protein DAMPs interacts with toll-like receptors on vascular cells to trigger transcription of pro-inflammatory factors *via* nuclear factor (NF)κB activation. This subsequently perpetuates complementary inflammatory cascades, like cytokines, chemokines, and adhesion molecules (Timmers et al., 2012). While the primary role of chemokines is to induce chemotaxis, together with adhesion molecules these pro-inflammatory factors mediate infiltration of neutrophils and monocytes into the injured myocardium. The presence of adhesion proteins on the injured endothelial surface of the myocardium leads to sequestration of free-flowing leukocytes followed by a slow-roll along the vessels in the course of blood circulation. The infiltrated neutrophils yield potent cytotoxic effects by releasing proteolytic enzymes, such as chymotrypsin and trypsin, to induce apoptosis in inflamed cells (Trevani et al., 1996). Monocytes also infiltrate into the injured myocardium along with neutrophils. The firm adhesion of monocytes to the endothelium is facilitated by chemokines, such as C-C motif chemokine ligand 2 (CCL2) and interleukin (IL)-8, which eventually leads to extravasation across the endothelium (Dewald et al., 2005). Recruitment of monocytes into the infarcted region can give rise to monocyte-derived macrophages with pro-inflammatory properties (Honold and Nahrendorf, 2018).

Secondary to the phagocytic role, the presence of macrophages in the infarcted myocardium is linked to an anti-inflammatory reparative phase. The transition from pro- to anti-inflammatory states is orchestrated between cardiac (e.g., cardiomyocytes, endothelial cells, fibroblasts) and inflammatory cells (e.g., neutrophils, monocytes, macrophages) (Honold and Nahrendorf, 2018). This multilayered signaling contributes to wound healing and scar formation to prevent cardiac rupture. The process is highly regulated; disturbance in the balance between the two inflammatory states may exacerbate injury and contribute to further heart dysfunction (Ong et al., 2018). As such, an overextended active inflammatory phase can lead to sustained tissue damage, improper tissue healing, such as defective scar formation, enlargement of the infarct tissue area, adverse cardiac remodeling, chamber dilatation, and ultimately decompensation in cardiac pumping ability. Currently, there are limited immunomodulatory or anti-inflammatory therapeutic regimes being implemented in clinical settings to treat acute MI. Targeting inflammatory cascades post-MI and initiating treatment regimes during the early phases of I/R injury have been recommended (Ong et al., 2018). However, if anti-inflammatory regimes are given too early, premature attenuation of the proliferative phase can occur, causing cardiac rupture or aneurysm formation.

## Prevention of Cardiovascular Events *Via* Anti-Inflammation

Although the primary function of the innate immune reaction is to promote homeostasis of tissue repair during post-reperfusion therapy, this may however act as a double-edged mechanism, resulting in further disease progression. In this respect, a dysregulated innate immune response has preconditioned a plethora of unnecessary pro-inflammatory releases. Acknowledgment of the double-edged mechanism of the inflammation in MI is clinically important as heart failure (HF) patients with reperfusion surgery have poor prognoses. This therefore presents medical practitioners with significant therapeutic challenges (Kadesjö et al., 2019). Results from clinical trials targeting inflammatory factors in MI are inconsistent and not always in line with initial hypotheses. An earlier prospective cohort study among individuals above 55 years old (*n* = 164,862) showed that NSAIDs increased the risk of a relapse in subjects with existing cardiovascular diseases, calling for careful drug prescription (Feenstra et al., 2002). Indeed, broad anti-inflammatory therapy may inhibit important repair pathways and negatively impact patients with MI (Huang and Frangogiannis, 2018). Conversely, inhibition of specific inflammatory modulators may present promise. For instance, canakinumab is an anti-IL1β biologic used to treat autoimmune diseases such as rheumatoid arthritis. In a randomized, double-blind trial, the Canakinumab Anti-inflammatory Thrombosis Outcome Study (CANTOS) tested the effects of three doses of canakinumab (50, 150, 300 mg) delivered subcutaneously every 3 months in 10,061 patients with a history of MI. Analysis of the primary study endpoints revealed that 150 mg reduced the relative risk of cardiovascular events and hospitalization by 15% compared to placebo (Ridker et al., 2017). In a dose-dependent manner and without affecting cholesterol levels, canakinumab treatment also reduced systemic inflammation biomarkers, such as high-sensitivity C-reactive protein (hsCRP) and IL-6, which have been associated with the presence of heart disease (Ridker et al., 2017; Ridker et al., 2018). However, the use of canakinumab was associated with a higher incidence of fatal infection (Ridker et al., 2017). In another study, The Cardiovascular Inflammation Reduction Trial (CIRT) examined the cardiovascular benefits of low-dose methotrexate, a broad-spectrum immunosuppressant drug, in MI patients (*n* = 5,500) (Ridker et al., 2019). Although CIRT had similar primary study endpoints to CANTOS, the use of methotrexate failed to reduce the risks of cardiovascular events and circulating inflammatory levels. Non-basal-cell skin cancers were also associated with methotrexate treatment in MI patients, hence calling for early termination of the study. While the CANTOS trial supported the therapeutic plausibility of targeting specific inflammatory pathways in MI, clinical implementation of anti-inflammatory agents in high-risk cardiovascular disease patients still requires further investigation until the risk–benefit trade-off has been clarified.

## Heterogeneity of Cardiac Macrophages in Myocardial Infarction

Macrophages are key regulators for cardiac inflammatory reactions in post I/R cardiac remodeling. Pro-inflammatory macrophages in the ischemic myocardium may originate from resident monocytes which are capable of switching their biological functions based on microenvironmental cues (Zhao et al., 2019). Indeed, an integrative computational model suggests activation of pro-inflammatory macrophages can be induced in the presence of specific cytokines such as tumor necrosis factor (TNF)α, interferon (IFN)γ, or hypoxic factors which are released by injured cellular components as innate immune responses (Zhao et al., 2019). The secretion of pro-inflammatory cytokines activates cardiac fibroblasts which in turn produce matrix proteases [matrix metalloproteinase (MMP)2, MMP9] in addition to other pro-inflammatory cytokines such as IL-1β and IL-6 (Ploeger et al., 2013). Proteases are pivotal in degrading the extracellular matrix to initiate tissue remodeling. The innate immune response of fibroblasts therefore provides a positive feedback loop, amplifying the pro-inflammatory microenvironment, to facilitate the clearance of necrotic cells within the ischemic and peri-ischemic regions (O’Rourke et al., 2019). During 5–7 days post-reperfusion, resident macrophages in the ischemic murine heart display a pro-reparative genetic signature (Mouton et al., 2018). These macrophages release anti-inflammatory cytokines, such as IL-10, which limit activation and proliferation of pro-inflammatory subtypes through the signal transducer and activator of transcription (STAT)3 pathway (Ploeger et al., 2013). Anti-inflammatory macrophages secrete numerous growth factors, such as vascular endothelial growth factor (VEGF) and transforming growth factor (TGF)β, that increase the capacity of matrix components to produce and deposit collagen for scar formation. PET/MRI in MI mice that received RNA interference treatment to target pro-inflammatory macrophages showed improvements in infarct inflammation and preserved LV function (Majmudar et al., 2013). Subsequent genomic and proteomic analyses revealed attenuation of the pro-inflammatory macrophage population in the infarcted region and elevated secretion of anti-inflammatory cytokines such as IL-10 and IL-6 (Majmudar et al., 2013). This suggests that prolonged inflammatory macrophage activity may exacerbate tissue remodeling post-infarction.

## Macrophage Polarization as a Therapeutic Target in Myocardial Infarction

The hyperactivity in pro-inflammatory macrophages can be regulated by the activation of α7nAChR which is highly expressed on the surface of inflammatory cells (Zhang et al., 2017). Examination in a murine model with lipopolysaccharide (LPS)-induced acute lung injury showed attenuation of inflammatory responses in the presence of an α7nAChR agonist (Wang et al., 2019). The anti-inflammatory action of α7nAChR was associated with protection against lung injury and polarization of macrophages with anti-inflammatory subtypes (Wang et al., 2019). The restorative benefit of the cholinergic modulation is also reported in MI animals where inhibition of ACh degradation with pyridostigmine showed dense macrophages with anti-inflammatory phenotypes in ischemic and peri-ischemic regions (Rocha et al., 2016; Bezerra et al., 2017). Interestingly, despite equivalent numbers of total macrophages counted in both vagal and non-vagal activated groups, more immunoreactive pro-inflammatory subtypes were observed in the non-stimulated animals (Rocha et al., 2016). This suggests an enhancement of macrophage plasticity, with anti-inflammatory predominance in the presence of cholinergic agents. Indeed, microglia cocultured with LPS endotoxins resulted in aggravated pro-inflammatory release of cytokines, such as IL-1 and IL-6, within the first few hours (Zhang et al., 2017). However, ACh treatment both pre- and post-LPS stimulation suppressed the pro-inflammatory cytokine release and increased production of anti-inflammatory cytokines, such as IL-4 and IL-10. The pro-inflammatory suppressive effect of ACh was inhibited in α7nAChR knockdown rats without affecting anti-inflammatory cytokine levels. It therefore seems that shifting macrophages toward anti-inflammatory subtypes by vagal activation alters the cellular milieu, by modulating pro-inflammatory release and maintaining the anti-/pro-inflammatory protein balance.

The anti-inflammatory mechanism of VNS remains unclear. One hypothesis is that vagal activation inhibits NFκB translocation and phosphorylation, therefore curtailing inflammatory signaling (Han et al., 2014). During acute MI, inflammatory signals (e.g., TNFα, IL-6) are released by the injured cells and bind to toll-like receptors to assist with the translocation and phosphorylation of NFκB, a transcriptional activator for inflammatory response (Han et al., 2014). It has also been postulated that α7nAChR activation triggers the CAP *via* Janus kinase (JAK)2-STAT3 signaling, a known negative regulator for inflammatory responses (De Jonge and Ulloa, 2007). Interestingly, *in vivo* studies reported elevated pro-inflammatory macrophages with activated STAT3 in hypertension, a known predisposing factor for MI (Loperena et al., 2018). Further, an *in vitro* study using endothelial cells revealed an activation of STAT3 due to an increased endothelial stretch (Loperena et al., 2018). Meanwhile, inhibition of STAT3 prevented the monocyte inflammatory response toward a dysfunctional endothelium (Loperena et al., 2018). Hence, it is pivotal to understand the mechanistic interventions of α7nAChR activation to enhance STAT3 activity in mediating anti-inflammatory roles in MI and other related conditions.

## Current Development in Vagal Neuromodulation Technique as a Therapy for Myocardial Infarction?

Modulation of vagal nerve activity has emerged as a potential non-pharmacological therapy for HF after MI. It is generally accepted that HF is associated with autonomic nervous system (ANS) imbalance, characterized by enhanced sympathetic tone and withdrawal of parasympathetic activity. Evidence in healthy humans shows that tVNS reduces muscle sympathetic nerve activity, thus suggesting the plausibility of the nerve modulation technique to restore imbalance in the ANS (Clancy et al., 2014). VNS/tVNS also appears to attenuate the progressive loss of ventricular function from post-ischemic cardiac remodeling (see Table 1 for a summary). For instance, chronic vagal stimulation (for 6 weeks) in post-MI rats showed improvement in LV end diastolic pressure and lower normalized biventricular weight compared to the untreated rats (Li et al., 2004). Similar observations were obtained from a combination therapy with metoprolol and chronic VNS in MI rats; improvements in LV function were associated with better prognosis in comparison to metoprolol alone (Li H. et al., 2019). Although, both studies reported no statistically significant changes in infarct size following VNS (Li et al., 2004; Li M. et al., 2019). Infarct size reduction has been consistently reported in other VNS-MI studies involving activation of α7nAChR (Chen et al., 2016; Kiss et al., 2017; Buchholz et al., 2018; Nuntaphum et al., 2018). The discrepancies could be attributed to different stimulation protocols and parameters (Uitterdijk et al., 2015; Chen et al., 2016; Buchholz et al., 2018; Nuntaphum et al., 2018).

**TABLE 1:** Studies on invasive vagal neuromodulation in animal models of myocardial infarction.

Invasive VNS						

Author	Species	*N*	MI model	Stimulation protocol	Stimulation parameters	Myocardial infarction outcomes
Nuntaphum et al., 2018	Swine	30	I/R	VNS applied on the left cervical vagal trunk at the onset of ischemia (60 min), continued until the end of reperfusion.	3.5 mA, 0.5 ms, 20 Hz	Reduction in infarct size, arrhythmia score, oxidative stress, apoptosis. Intact contralateral vagal efferent required to provide better cardioprotection.
Chen et al., 2016	Dog	30	I/R	VNS applied on the left cervical vagal trunk for 120 min during reperfusion.	20 Hz, 0.1 ms, 80% below voltage threshold.	Reduction in infarct size, myocardial neutrophil infiltration, inhibition of oxidative stress and apoptosis.
Uitterdijk et al., 2015	Swine	23	I/R	VNS applied on left vagal nerve 5 min prior to reperfusion and continued until 15 min of reperfusion.	10 mA, 0.3 ms, 25 Hz	Reduction in infarct size, area of no-reflow, neutrophil and macrophages infiltration in the infarct area. NO-synthase activity was required for VNS cardioprotection.
Li M. et al., 2019	Rat	100	CHF	VNS applied on right cervical vagus nerve with a duty cycle of 16.7% (10 s on/50 s off) for 24 h and last for 6 weeks.	0.2 ms, 20 Hz	VNS + metoprolol treatment (VSM) improved 50-day survival rate, cardiac index and maximum left ventricular pressure (LV + dp/dt_max_). VSM also reduced heart rate, normalized biventricular weight, left ventricular end diastolic pressure (LVEDP), right arterial pressure, plasma norepinephrine, epinephrine and brain natriuretic peptide (BNP). VNS exerts its cardioprotection independent of anti-beta-adrenergic pathway.
Li et al., 2004	Rat	85	CHF	VNS applied on right cervical vagus nerve for 10 s every minute for 6 weeks.	0.2 ms, 20 Hz, 0.1–0.13 mA (adjusted for each rat)	VNS improved 140-days survival rate, LV + dp/dt_max_ and also reduced LVEDP, normalized biventricular weight, plasma norepinephrine and BNP.
Buchholz et al., 2018	Rat	59	I/R	Right cervical vagus nerve was stimulated for 10 min prior to ischemia or the initial 10 min during reperfusion	0.1 ms, 10 Hz	Both preischemic VNS and reperfusion VNS reduced heart rate and infarct size. Preischemic VNS provides cardioprotection via Akt/GSK-3β muscarinic pathway while reperfusion VS via α7nAchR activation.
Kiss et al., 2017	Rat	76	I/R	Right cervical nerve was stimulated for 2 h during reperfusion.	0.5 ms, 15 Hz, 0.1–1 mA	VNS reduced infarct size and reversed the upregulation of arginase activity induced by IR. These effects were abolished by α7nAChR blockade.

Perhaps unsurprisingly, clinical trials of VNS also report contradictory therapeutic endpoints (see Table 2 for a summary). A 6-month pilot study of VNS (CardioFit^TM^) in patients with ventricular dysfunction (*n* = 8) found significant reductions in cardiac end systolic volume as well as improvement in New York Heart Association (NYHA) class function (Schwartz et al., 2008). De Ferrari et al. (2011) extended this study into a multicenter, two-stage trial (*n* = 32) where similar data collection points were combined with an optional 1-year follow-up. VNS successfully improved patient NYHA class, quality of life, exercise ability, LV systolic volume, as well as LVEF (De Ferrari et al., 2011). The Autonomic Neural regulation THerapy to Enhance Myocardial function in Heart Failure (ANTHEM-HF) study assessed the effects of therapy on LV structure and function in patients with chronic stable HF. Stimulating either the left or the right side of the vagus nerve in HF patients significantly improved cardiac contractility as measured from LVEF and pro-brain natriuretic peptide (BNP) (a plasma HF marker) (Premchand et al., 2016). However, a randomized sham control trial [Neural Cardiac Therapy for Heart Failure (NECTAR-HF)] failed to demonstrate any significant changes in the endpoint measures after 6 months of right vagal stimulation (Zannad et al., 2015). Additionally, a multinational randomized trial [Increase of Vagal Tone in Heart Failure (INOVATE-HF)] showed the risk of death or events among HF patients was not improved in the VNS-treated group (Gold et al., 2016). Despite conflicting cardiac functions as primary endpoints, VNS studies agreed that long-term therapy improved overall quality life. Inconclusive outcomes from these VNS clinical trials are potentially due to heterogeneity in study samples where different patient groups have specific clinical presentations and therapeutic needs. Differences in outcomes may also be compounded by the VNS protocols that seem to vary from study to study (Li M. et al., 2019).

**TABLE 2:** Clinical trials on invasive vagal neuromodulation in patients with heart failure.

Clinical trials						

Author	Acronym	*N*	Sampling group	Stimulation protocol	Stimulation parameters	Cardioprotective outcomes
Schwartz et al., 2008		8	(i) 18–75 years old (ii) Left ventricular ejection fraction < 35% (iii) History of CHF in NYHA class II–III (iv) Screening Holter ECG average 24 h heart rate ≥65 beats/min (v) Occurrence of ≥2 episodes of heart rate ≥80 without physical exercise and capable of 6 min walk test.	Stimulation started 2–3 weeks after *Cardiofit* implantation. 3–4 visits/week were performed during the 3 weeks titration phase to slowly increase the current until discomfort was reported. Current was set below the point where discomfort was reported. Follow up sessions were carried out 1,3 and 6 months after optimization to evaluate patients.	Delivery of 1 ms pulse/beat 70 ms after R wave with an amplitude of 2 mAmp. Intermittent pulse application with 2–10 s ON time followed by 6–30 s OFF time.	VNS was reported to be safe and tolerable on patients with mild side effects. VNS also significantly improved patient’s NYHA class, Minnesota quality of life score and left ventricular end systolic volume (LVESV).
Premchand et al., 2016	ANTHEM-HF	60	NYHA class II-III heart failure patients with ≤40% of left ventricular ejection fraction (LVEF).	Patients were implanted with VNS device (Demipulse Model 103 pulse generator and PerenniaFLEX Model 304 lead, Cyberonics, Houston, TX, United States) with 1:1 randomized lead placement to either left or right cervical vagus nerve. During the 10 weeks titration phase, stimulation parameters were systematically adjusted. During the 6 months follow up period, 49 patients (23 left sided, 26 right sided) consented to take part in extended follow up visits at 9 and 12 months.	250 μs, 10 Hz, 2.0 ± 0.6 mA	VNS was safe and well-tolerated in patients with minimal side effects. VNS significantly improved patient’s LVEF and LVESD at 6 and 12 months with the exception of LVESV improving at 12 months. VNS also improved NYHA class, 6 min walk distance and scores on the Minnesota Living with Heart Failure Questionnaire.
De Ferrari et al., 2017	NECTAR-HF	96	NYHA class II or III patients with LVEF ≤35% and LV end diastolic dimension (LVEDD) ≥55 mm.	In the first phase, patients were implanted with Boston Scientific VNS system and randomized with 2:1 ratio into group receiving standard medical treatment with active VNS vs. group receiving standard medical treatment with inactive VNS for 6 months. Inactive VNS was activated after 6 months. In the second phase, patients were evaluated once again after 18 months.	20 Hz, 300 μs0, 10 s ON time followed by 50 s OFF time, ≤4 mA.	VNS failed to show significant improvement on cardiac function in terms on LVESV, LVESD, and LVEF. However, VNS was able to improve NYHA class for all patients. Observation of heart rate effect using heat map technique revealed that patients in NECTAR-HF had significantly less recruitment of fibers involved in heart rate changes.
Gold et al., 2016	INOVATE-HF	707	NYHA class III patients with LVEF ≤%, LVEDD of 50–80 mm and QRS duration <120 ms.	Patients were implanted with *CardioFit* system and underwent 3:2 ratio of randomization into group receiving standard medical treatment + VNS vs. standard medical treatment only. Follow-up evaluations were carried out at 3 month intervals during the 18 months post-implant, followed by 6 month intervals for overall status.	3.9 ± 1.0 mA	VNS did not reduced mortality of patients and did not induce reverse remodeling. Nonetheless, VNS significantly improved NYHA class, 6 min walk duration and quality of life measures.

Activation of the vagus nerve may be achieved non-invasively through electrical stimulation of select auricular dermatomes with vagal afferent innervation (Peuker and Filler, 2002). Indeed, the vagus nerve has an auricular branch, thought to innervate the tragus, concha, and cymba concha, to which electrical stimulation can be applied. This involves using a transcutaneous electrical nerve stimulator (TENS) machine connected to auricular clips to deliver small, pain-free, just perceptible electrical impulses to the auricular dermatome of interest (usually the tragus, concha, or cymba concha). As such, tVNS could be useful for many MI patients especially as it is painless, inexpensive (the cost of a TENS machine), simple to administer (i.e., can be performed in the comfort of one’s own home), well-tolerated, and highly convenient, particularly for patients who have restrictions on pharmacological therapies (Wang et al., 2015).

Applying low-level tragus stimulation (20 Hz, 1 ms, duty cycle of 5 s on 5 s off) in dogs with healed MI for 90 days attenuated ventricular remodeling, as recognized by a smaller infarct size and better cardiac contractile and diastolic functions (Wang et al., 2014). Normally, in post-MI (without any stimulation or sham control), cardiac tissue starts to develop scars and the level of fibrosis is influenced by enhanced sympathetic nerve activity (Wang et al., 2014). However, the group that received low-level tragus stimulation exhibited reductions in plasma norepinephrine (NE) levels, suggesting that central sympathoinhibition may be responsible for the amelioration of the post-MI remodeling (Wang et al., 2014). In a study by Zhou et al. (2019) on Dahl salt-sensitive rats, inflammatory responses within the LV were successfully abrogated following 4 weeks of tVNS (20 Hz, 0.2 ms, 2 mA). In this model, cardiac injury on the LV signifies maladaptive physiological reactions due to prolonged elevations in blood pressure (Zhou et al., 2019). However, non-invasive VNS prevented hypertension and deteriorations in LV diastolic pumping function (Zhou et al., 2019).

Despite promising animal studies, evidence to support the cardioprotective effects of auricular VNS in real MI patients is still lacking. However, initial evidence comes from a trial in MI patients with impaired LV contractility during preoperative management where tVNS was delivered for 15 min and repeated for 10 consecutive days (Zamotrinsky et al., 2001). The study reported an increased density of cardiac noradrenergic plexuses, improved hemodynamic properties, and reduced incidence of transient heart failure during the post-operative period. Another study was performed in patients who underwent balloon angioplasty within 12 h of MI symptom onset (Yu et al., 2017). Low-level tVNS treatment (20 Hz, 1 mA) applied on the right tragus for 2 h immediately after the procedure successfully attenuated MI reperfusion injury. The cardioprotective characterizations were recorded after 7 days where blood inflammatory markers, incidence of ventricular arrhythmia, and echocardiography cardiac functions were improved. In another study, similar tVNS parameters (20 Hz, 1 mA) were tested in patients with diastolic dysfunction (*n* = 10) (Stavrakis et al., 2017). One hour of tVNS therapy successfully improved diastolic function (increased by 2.7%) and was associated with a shift in HRV index toward vagal predominance (Stavrakis et al., 2017). These early works (see Table 3 for a summary) therefore suggest that tVNS could provide cardioprotective effects for patients with MI, reinforcing the need for considerably more research in this area.

**TABLE 3:** Studies of cardioprotection by transcutaneous VNS.

Transcutaneous VNS						

Author	Species	*N*	MI model	Stimulation protocol	Stimulation parameters	Myocardial infarction/cardioprotective outcomes
Wang et al., 2014	Dog	30	Healed MI	Low level tragus stimulation (LLTS) was applied at the bilateral tragus of the external auditory canal in conscious dogs for 4 h (7–9 am, 4–6 pm).	20 Hz, 1 ms, 5 s on and 5 s off duty cycle	Treatment of LLTS significantly attenuated left atrial (LA) and left ventricular (LV) dilatation, improved LV contractile and diastolic function and also significantly reduced infarct size and cardiac fibrosis. Protein expressions of transforming growth factor β1 (TGF-β1), matrix metallopeptidase 9 (MMP-9), collagen I and collagen III were significantly attenuated through treatment of LLTS. Plasma high specific C-reactive protein (hs-CRP), norepinephrine (NE) and N-terminal pro B-type natriuretic peptide (NT-proBNP) were also significantly reduced in LLTS group.
Zhou et al., 2019	Rat	48	Heart failure with preserved ejection fraction (HFpEF).	LLTS was applied at the auricular concha region in anesthesized rats 30 min daily for 4 weeks.	20 Hz, 0.2 ms, 2 mA	Treatment of LLTS significantly attenuated the elevation of blood pressure, reduced LV hypertrophy, inhibited the deterioration of LV diastolic function and attenuated LV inflammatory cell infiltration and fibrosis compared with sham control. Likewise, improvement of LV circumferential strain was also observed through treatment with LLTS. Expression of pro-inflammatory and pro-fibrotic genes were downregulated in LLTS group suggesting that LLTS preserved LV function possibly through the suppression of myocardial pro-inflammatory and pro-fibrotic gene expression.
Zamotrinsky et al., 2001	Human	18	Coronary artery disease (CAD) with stable angina pectoris	Auricular vagus nerve was stimulated using acupuncture needle for 10 stimulation procedures that lasted for 15 min and repeated for 10 days.	3 Hz, 1.5 ms, 0.2–1.5 mA	VNS treatment conveyed an anti-anginal effect and reduced blood pressure and heart rate. Improvement of LVEF and left ventricular diastolic filling were also observed in VNS group. Electrocardiograms of VNS group reported a shortening of QRS and QT duration, no changes on PQ interval and an improvement of T-wave. VNS treatment also attenuated the progression of heart failure in patients.
Yu et al., 2017	Human	95	ST-elevation myocardial infarction (STEMI)	STEMI patients that underwent percutaneous coronary intervention (PCI) were given LLTS at right tragus for 2 h after balloon dilatation.	20 Hz, 1 ms, 50% below threshold, duty cycle 5 s on and 5 s off	Treatment of LLTS significantly attenuated the occurrence of reperfusion associated ventricular arrhythmia during first 24 h. The area under curve for level of creatine kinase-MB and myoglobin were significantly reduced by LLTS 72 h after reperfusion. In addition, level of NT-proBNP was significantly reduced by LLTS at 24 h and 7 days post-reperfusion. Significant improvements of inflammation, LVEF and wall motion index were observed as well in LLTS group.
Tran et al., 2019	Human	24	Diastolic dysfunction and preserved LVEF	LLTS was applied on patients for two sessions. Each session lasted for 1 h and was separated by at least 1 day to 1 week.	20 Hz, 200 μs, 1 mA	LLTS treatment improved left ventricular global longitudinal strain and induced favorable alteration of heart rate variability (HRV) frequency domain parameters in terms of high frequency (HF), low frequency (LF) and LF/HF ratio.

## Perspectives and Future Studies

### How Does Auricular Stimulation Activate the Vagus Nerve?

It is important to note that although other nerves innervate the auricle, e.g., the great auricular nerve and the auriculotemporal nerve, they are often underappreciated and findings are based on one research study (Peuker and Filler, 2002; Burger and Verkuil, 2018). Our previous neuro tracing study in rats showed dense termination of nerve afferents from the tragus into the ipsilateral dorsal region of the upper cervical spinal cord between C2 and C4 (Mahadi et al., 2019). Further neurophysiology recordings from the central sympathetic trunk showed acute reductions in sympathetic activity after tragus stimulation; equivalent as per reported in healthy humans (Clancy et al., 2014; Mahadi et al., 2019). Interestingly, the inhibitory effect persisted despite cervical vagal sectioning ipsilateral to the stimulation point. These inhibitory effects then became attenuated following an incision on the upper cervical afferent nerve roots. As a result, this prompts for careful interpretation of VNS, particularly with the transcutaneous method.

**FIGURE 1:**
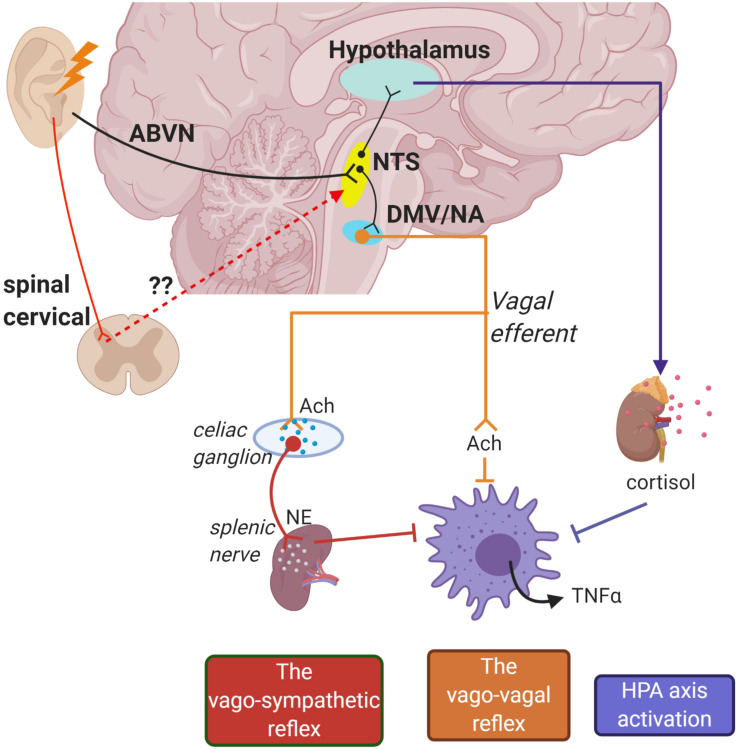
Schematic of potential pathways for how auricular stimulation could provide cardioprotection against cardiac remodeling in myocardial infarction (MI). Electrical stimuli applied on the auricle directly activate the nucleus tractus solitarius (NTS) through the auricular branch of the vagus nerve (ABVN) afferents. The NTS is also potentially activated in secondary order from somatosensory neurons in the upper cervical spinal cord. The NTS is the central control for autonomic functions and activates the hypothalamus–pituitary–adrenal axis to release anti-inflammatory cortisol. Initiation of action potentials in the NTS also sends signals to cell bodies in the dorsal motor nucleus of the vagus (DMV) and nucleus accumbens (NA) which give rise to vago-vagal and vago-sympathetic reflexes.

Intriguingly, neuroimaging studies repeatedly reported involvement of vagal associated regions in the brain stem which are ipsilateral to the stimulation site in the ear, such as the nucleus tractus solitarius (NTS), spinal trigeminal nucleus, and locus coeruleus (Yakunina et al., 2017). This unlocks an avenue for discussion regarding whether auricular vagal neurostimulation is subserved by the deep and superficial dorsal horn neural activity: perceived from “heat/electrical” signal sensations triggered from the skin. Dorsal horn neurons are typically associated with conveying sensory information from the skin and internal organs to the spinothalamic tract for pain signaling (Todd, 2010). Of note, auricular nerve stimulation was demonstrated to modulate sympathetic tone potentially through dorsal horn neural connectivity (Clancy et al., 2014; Mahadi et al., 2019). Despite not being widely recognized, the functionality of neurons in the cervical dorsal horn can also be observed in autonomic control of the cardiovascular system (Todd, 2010). In fact, spinal cord stimulation (SCS) at the lumbar region has been linked with increased vagal tone measured from HRV in patients with severe pain (Goudman et al., 2019). In addition, lumbar SCS demonstrated α7nAChR-mediated microglia inhibition in rabbits with spinal cord ischemia injury (Li H. et al., 2019). Anterograde tracing in rats demonstrates that the neurons of the superficial laminae of the dorsal horn terminate in the NTS which is an autonomic-vagal relay center (Potts et al., 2002). Hence, it is worth considering that the therapeutic benefits elicited by auricular neurostimulation may also be mediated by spinal activation (Figure 1).

### Proposed Neural Mechanisms for the Cholinergic Anti-Inflammatory Pathway

The exact interaction between the transcutaneous vagus nerve and neuro-immune axis remains to be elucidated, and several mechanisms have been proposed (Bonaz et al., 2017). One theory proposes that vagal afferent signaling propagates to the hypothalamus–pituitary–adrenal (HPA) axis. During peripheral inflammation, a surge of IL-1β occurs in the brain (Hosoi et al., 2000). Vagal afferents receive input from glomus cells of paraganglia which have a binding affinity for inflammatory cytokine IL-1β (Goehler et al., 1997). This activates NTS noradrenergic A2 neurons and subsequently relays the information to corticotrophin-releasing factor (CRF) neurons located in the parvo-cellular region of the paraventricular nucleus of the hypothalamus (PVH). CRF stimulates the pituitary gland to secrete adrenocorticotropic hormone (ACTH) which in turn induces adrenal production of glucocorticoids for the inhibition of peripheral inflammation. Electrical stimulation of vagal afferents in immune challenged rats upregulates CRF mRNA expression in the hypothalamus, increasing the production of plasma ACTH and corticosterone (Hosoi et al., 2000; De Herdt et al., 2009). In healthy individuals, targeting vagal innervation in the cymba conchae prevented a decline in salivary cortisol levels, a glucocorticoid stress hormone, suggesting activation of the HPA axis during tVNS (Warren et al., 2019).

Other proposed pathways involve the activation of vagal efferents. Earlier work by Borovikova et al. (2000) demonstrated that bilateral stimulation to the distal end of a vagotomized nerve trunk suppressed systemic inflammatory responses in endotoxemic rats. ACh released by the nerve endings inhibited pro-inflammatory cytokine release by human macrophages *in vitro*. The neuro-immune benefits elicited from vagal efferent activation were silenced in α7-cholinergic receptor-deficient mice during endotoxemia (Wang et al., 2003). Thenceforth, the immune-vagal neural interaction was commonly referred to as the CAP. However, synaptic connection from the vagus nerve to the spleen, a lymphoid organ that is primarily involved in the production of immune cells, has been proven sparse (Bratton et al., 2012). Surgical ablation of the splenic nerve in rats with endotoxemia abrogated the downregulation of pro-inflammatory cytokines with VNS, highlighting its functional role in mediating the CAP (Rosas-Ballina et al., 2008). Similar observations were obtained in catecholamine-depleted rats, denoting the synergistic effects of sympathetic nervous activity in the CAP. Electrical stimulation of the splenic nerve controls systemic inflammation, prevents septic shock, and restores neuromodulation benefit in α7nAChR-knockout mice (Vida et al., 2011a). However, pharmacological inhibition of β2-adrenoreceptors in endotoxemia rats prevented the anti-inflammatory potential of splenic nerve stimulation (Vida et al., 2011b). As such, the splenic nerve may act as an intermediary link between both autonomic nervous activities and the CAP response (Figure 1). Although controversial, some studies suggest sole sympathetic-splenic nerve input into the “cholinergic anti-inflammatory reflex” without the influence of vagus nerve activation (Martelli et al., 2014, 2019). While this has been shown in an endotoxemia sepsis model, this does not fully account for the effects of vagal neuromodulation observed from other experimental models, e.g., heart failure (Kiss et al., 2017).

### Investigating the Effects of Transcutaneous Vagus Nerve Stimulation in Individuals With Myocardial Infarction

Collectively, there is considerable preclinical evidence suggesting that VNS/tVNS is associated with beneficial cardioprotective effects (Wang et al., 2014; Zhou et al., 2019). However, the translational benefit of VNS in human trials has proven difficult (Statz and Olshansky, 2019). Hence, finding a good foundation for VNS/tVNS research which further examines the potential impact of this novel therapy on the lives and health of individuals with cardiovascular conditions is warranted.

Prior work in patient cohorts has tended to examine the effects of tVNS administered acutely and for short time periods (i.e., 7 days). Therefore, future work should explore the magnitude of the cardioprotective impacts of tVNS delivered over longer time frames (e.g., 1, 2, 6 months, etc.). Of course, in order to explore the contribution of placebo effects to improvements associated with active stimulation, control conditions should be carefully considered. This is an important point given that tVNS is perceptible, therefore making it difficult to undertake double-blinding in randomized control trials. Furthermore, due to the recent controversy in the literature surrounding the innervation of nerves in the external ear (Badran et al., 2018; Burger and Verkuil, 2018; Mahadi et al., 2019), it is even more imperative to determine a valid control for tVNS in cardiovascular research. Control conditions have tended to comprise: (1) turning off the device that delivers the stimulation without the participant’s knowledge while positioned on the same part of the external ear as used during active stimulation (Clancy et al., 2014; Antonino et al., 2017; Bretherton et al., 2019) or (2) delivering active stimulation to a part of the external ear thought not to be innervated by the vagus nerve, e.g., the earlobe (Antonino et al., 2017; Badran et al., 2018; Burger and Verkuil, 2018). Indeed, active stimulation is associated with superior effects compared to both sham stimulation techniques (Clancy et al., 2014; Antonino et al., 2017; Badran et al., 2018; Burger and Verkuil, 2018; Bretherton et al., 2019). Perhaps future work should also incorporate an arm in which participants receive conventional MI treatments [e.g., β-blockers, NSAIDs, and angiotensin-converting enzyme (ACE) inhibitors] in order to compare the efficacy and safety of tVNS with current medical interventions.

It would also be prudent for future work to reflect on which outcome measures would be of interest in cardiovascular conditions. This is particularly crucial for clinicians if tVNS is to be considered as a potential (adjunct) treatment modality. Outcome measures could include infarct size, cardiac contractile, diastolic and LV function, NYHA classification, mortality rate, hospital and general practitioner (GP) admissions, length of hospital stays, and medication consumption. As HF is characterized by impairments in undertaking physical activity, usually accompanied with reductions in quality of life, measures which provide insight into the real-life consequences of the condition would be valuable especially for patients. This could include heart rate, oxygen uptake, carbon dioxide output, ventilatory equivalent, respiratory rate, and anaerobic threshold during a ramping treadmill exercise test. Questionnaires, such as the Minnesota Living with Heart Failure questionnaire and the Godin Leisure Time Exercise Questionnaire, could also be used to provide subjective measures of health-related quality of life and physical activity levels.

Due to the inflammatory responses inherent in MI, plasma NE, pro-inflammatory cytokines, including TNFα, IL-1, and IL-6, and anti-inflammatory cytokines such as IL-4 and IL-10 would be worthwhile measuring. Indeed, these data could generate greater understanding about what happens at the molecular level in patients with MI when using tVNS (Stavrakis et al., 2017). As autonomic balance is also impaired in patients with coronary artery disease, measures of autonomic function such as HRV, baroreceptor reflex sensitivity, and sympathetic nerve outflow (derived by microneurography in the peroneal nerve) could also be incorporated. Functional brain imaging techniques may also be a useful addition, especially if a mechanistic account of tVNS in an MI patient cohort is a key objective.

## Conclusion

**FIGURE 2:**
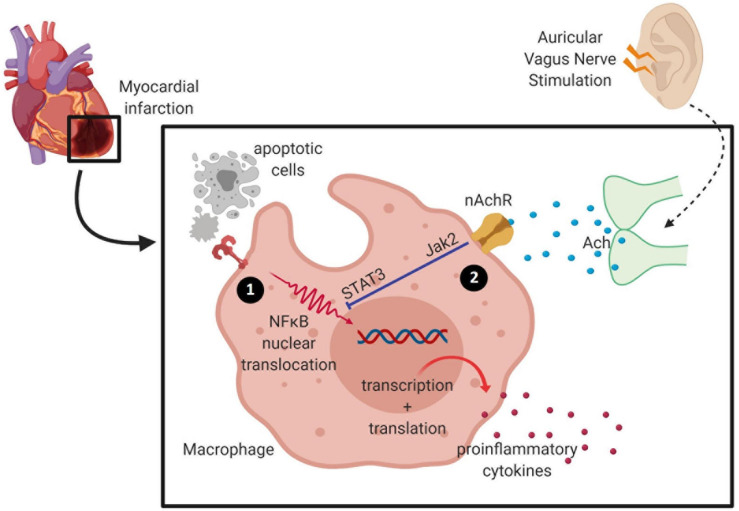
Activation of the cholinergic anti-inflammatory pathway inhibits pro-inflammatory release by macrophage. (1) During ischemia, hypoxia-activating factors are released by injured cells and cause activation of the nuclear factor (NF)-κB signaling pathway to induce pro-inflammatory cytokine release. Cardiac macrophages undergo phenotypic and metabolic reprogramming within a week of myocardial infarction (MI)—from high pro-inflammatory activity into a pro-reparative signature. (2) Acetylcholine released from the vagus nerve terminates in α7 nicotinic acetylcholine receptors (α7nAChR) expressed on macrophages, and these inhibit the release of pro-inflammatory cytokines.

Despite inconclusive data from clinical trials, it is becoming evident that electrical stimulation of the vagal nerve can elicit cardioprotective effects against adverse cardiac remodeling in animals. The cardioprotective effects of VNS involve activation of the CAP which may reprogram and rebalance the production of inflammatory cytokines (Figure 2). However, recent findings suggest that other neural pathways such as cervical spinal activation may be involved in auricular tVNS (Mahadi et al., 2019). It remains unknown how tVNS may activate the CAP in cardiac macrophages similar to that shown in invasive VNS. Combining molecular research with translational work investigating the cardioprotective effects of tVNS in patient samples will aid with generating an account of how this stimulation can improve the health of these patients, enabling them to live a more fulfilling life.

## Author Contributions

CHC and BB contributed to writing the original draft preparation. SZ contributed to writing, reviewing, and editing. SD and JD contributed to supervision and writing, reviewing, and editing. MM contributed to conceptualization, writing the original draft preparation, and visualization. All authors contributed to the article and approved the submitted version.

## Conflict of Interest

The authors declare that the research was conducted in the absence of any commercial or financial relationships that could be construed as a potential conflict of interest.

## References

[B1] Antonino D. Teixeira A. L. Maia-Lopes P. M. Souza M. C. Sabino-Carvalho J. L. Murray A. R. (2017). Non-invasive vagus nerve stimulation acutely improves spontaneous cardiac baroreflex sensitivity in healthy young men: a randomized placebo-controlled trial. *Brain Stimul.* 10 875–881. 10.1016/j.brs.2017.05.006 28566194

[B2] Badran B. W. Brown J. C. Dowdle L. T. Mithoefer O. J. LaBate N. T. Coatsworth J. (2018). Tragus or cymba conchae? Investigating the anatomical foundation of transcutaneous auricular vagus nerve stimulation (taVNS). *Brain Stimul.* 11:947. 10.1016/j.brs.2018.06.003 29895444 PMC6607436

[B3] Benjamin E. J. Virani S. S. Callaway C. W. Chamberlain A. M. Chang A. R. Cheng S. (2018). Heart disease and stroke statistics—2018 update: a report from the American heart association. *Circulation* 137 e67–e492. 29386200 10.1161/CIR.0000000000000558

[B4] Bezerra O. C. França C. M. Rocha J. A. Neves G. A. Souza P. R. M. Gomes M. T. (2017). Cholinergic stimulation improves oxidative stress and inflammation in experimental myocardial infarction. *Sci. Rep.* 7:13687. 10.1038/s41598-017-14021-8 PMC5651932 29057895

[B5] Bonaz B. Sinniger V. Pellissier S. (2017). The vagus nerve in the neuro-immune axis: implications in the pathology of the gastrointestinal tract. *Front. Immunol.* 8:1452. 10.3389/fimmu.2017.01452 29163522 PMC5673632

[B6] Borovikova L. V. Ivanova S. Zhang M. Yang H. Botchkina G. I. Watkins L. R. (2000). Vagus nerve stimulation attenuates the systemic inflammatory response to endotoxin. *Nature* 405:458. 10.1038/35013070 10839541

[B7] Bratton B. Martelli D. McKinley M. Trevaks D. Anderson C. McAllen R. (2012). Neural regulation of inflammation: no neural connection from the vagus to splenic sympathetic neurons. *Exp. Physiol.* 97 1180–1185. 10.1113/expphysiol.2011.061531 22247284

[B8] Bretherton B. Atkinson L. Murray A. Clancy J. Deuchars S. Deuchars J. (2019). Effects of transcutaneous vagus nerve stimulation in individuals aged 55 years or above: potential benefits of daily stimulation. *Aging* 11 4836–4857. 10.18632/aging.102074 31358702 PMC6682519

[B9] Buchholz B. Kelly J. Muñoz M. Bernatené E. A. Méndez Diodati N. González Maglio D. H. (2018). Vagal stimulation mimics preconditioning and postconditioning of ischemic myocardium in mice by activating different protection mechanisms. *Am. J. Physiol. Heart Circ. Physiol.* 314 H1289–H1297. 29631370 10.1152/ajpheart.00286.2017

[B10] Burger A. M. Verkuil B. (2018). Transcutaneous nerve stimulation via the tragus: are we really stimulating the vagus nerve? *Brain Stimul.* 11 945–946. 10.1016/j.brs.2018.03.018 29661599

[B11] Chen M. Zhou X. Yu L. Liu Q. Sheng X. Wang Z. (2016). Low-level vagus nerve stimulation attenuates myocardial ischemic reperfusion injury by antioxidative stress and antiapoptosis reactions in canines. *J. Cardiovasc. Electrophysiol.* 27 224–231. 10.1111/jce.12850 26546374

[B12] Clancy J. A. Mary D. A. Witte K. K. Greenwood J. P. Deuchars S. A. Deuchars J. (2014). Non-invasive vagus nerve stimulation in healthy humans reduces sympathetic nerve activity. *Brain Stimul.* 7 871–877. 10.1016/j.brs.2014.07.031 25164906

[B13] De Ferrari G. M. Crijns H. J. Borggrefe M. Milasinovic G. Smid J. Zabel M. (2011). Chronic vagus nerve stimulation: a new and promising therapeutic approach for chronic heart failure. *Eur. Heart J.* 32 847–855. 10.1093/eurheartj/ehq391 21030409

[B14] De Ferrari G. M. Stolen C. Tuinenburg A. E. Wright D. J. Brugada J. Butter C. (2017). Long-term vagal stimulation for heart failure: Eighteen month results from the NEural Cardiac TherApy foR Heart Failure (NECTAR-HF) trial. *Int. J. Cardiol.* 244 229–234. 10.1016/j.ijcard.2017.06.036 28663046

[B15] De Herdt V. Puimège L. De Waele J. Raedt R. Wyckhuys T. El Tahry R. (2009). Increased rat serum corticosterone suggests immunomodulation by stimulation of the vagal nerve. *J. Neuroimmunol.* 212 102–105. 10.1016/j.jneuroim.2009.04.013 19446345

[B16] De Jonge W. Ulloa L. (2007). The alpha7 nicotinic acetylcholine receptor as a pharmacological target for inflammation. *Br. J. Pharmacol.* 151 915–929. 10.1038/sj.bjp.0707264 17502850 PMC2042938

[B17] Dewald O. Zymek P. Winkelmann K. Koerting A. Ren G. Abou-Khamis T. (2005). CCL2/monocyte chemoattractant protein-1 regulates inflammatory responses critical to healing myocardial infarcts. *Circ. Res.* 96 881–889. 10.1161/01.res.0000163017.13772.3a 15774854

[B18] Feenstra J. Heerdink E. R. Grobbee D. E. Stricker B. H. C. (2002). Association of nonsteroidal anti-inflammatory drugs with first occurrence of heart failure and with relapsing heart failure: the Rotterdam Study. *Arch. Intern. Med.* 162 265–270. 10.1001/archinte.162.3.265 11822918

[B19] Goehler L. E. Relton J. K. Dripps D. Kiechle R. Tartaglia N. Maier S. F. (1997). Vagal paraganglia bind biotinylated interleukin-1 receptor antagonist: a possible mechanism for immune-to-brain communication. *Brain Res. Bull.* 43 357–364. 10.1016/s0361-9230(97)00020-8 9227848

[B20] Gold M. R. Van Veldhuisen D. J. Hauptman P. J. Borggrefe M. Kubo S. H. Lieberman R. A. (2016). Vagus nerve stimulation for the treatment of heart failure: the INOVATE-HF trial. *J. Am. College Cardiol.* 68 149–158. 10.1016/j.jacc.2016.03.525 27058909

[B21] Goudman L. Brouns R. Linderoth B. Moens M. (2019). Effects of spinal cord stimulation on heart rate variability in patients with failed back surgery syndrome. *PLoS One* 14:e0219076. 10.1371/journal.pone.0219076 31260496 PMC6602188

[B22] Han Z. Shen F. He Y. Degos V. Camus M. Maze M. (2014). Activation of α-7 nicotinic acetylcholine receptor reduces ischemic stroke injury through reduction of pro-inflammatory macrophages and oxidative stress. *PLoS One* 9:e105711. 10.1371/journal.pone.0105711 25157794 PMC4144901

[B23] Honold L. Nahrendorf M. (2018). Resident and monocyte-derived macrophages in cardiovascular disease. *Circ. Res.* 122 113–127. 10.1161/circresaha.117.311071 29301844 PMC5777215

[B24] Hosoi T. Okuma Y. Nomura Y. (2000). Electrical stimulation of afferent vagus nerve induces IL-1β expression in the brain and activates HPA axis. *Am. J. Physiol. Integr. Comp. Physiol.* 279 R141–R147. 10.1152/ajpregu.2000.279.1.R141 10896875

[B25] Huang S. Frangogiannis N. G. (2018). Anti-inflammatory therapies in myocardial infarction: failures, hopes and challenges. *Br. J. Pharmacol.* 175 1377–1400. 10.1111/bph.14155 29394499 PMC5901181

[B26] Huang S.-P. Wen Y.-C. Huang S.-T. Lin C.-W. Wang T.-D. Hsiao F.-Y. (2019). Nonsteroidal anti-inflammatory drugs and risk of first hospitalization for heart failure in patients with no history of heart failure: a population-based case-crossover study. *Drug Saf.* 42 67–75. 10.1007/s40264-018-0720-9 30232741

[B27] Kadesjö E. Roos A. Siddiqui A. Desta L. Lundbäck M. Holzmann M. J. (2019). Acute versus chronic myocardial injury and long-term outcomes. *Heart* 105:315036. 10.1136/heartjnl-2019-315036 31337668

[B28] Kiss A. Tratsiakovich Y. Mahdi A. Yang J. Gonon A. Podesser B. (2017). Vagal nerve stimulation reduces infarct size via a mechanism involving the alpha-7 nicotinic acetylcholine receptor and downregulation of cardiac and vascular arginase. *Acta Physiol.* 221 174–181. 10.1111/apha.12861 28238218

[B29] Li H. Dong X. Cheng W. Jin M. Zheng D. (2019). Neuroprotective mechanism involved in spinal cord stimulation postconditioning. *J. Thorac. Cardiovasc. Surg.* 159 813.e1–824.e1. 31030961 10.1016/j.jtcvs.2019.03.048

[B30] Li M. Zheng C. Kawada T. Inagaki M. Uemura K. Sugimachi M. (2019). Chronic vagal nerve stimulation exerts additional beneficial effects on the beta-blocker-treated failing heart. *J. Physiol. Sci.* 69 295–303. 10.1007/s12576-018-0646-0 30414045 PMC10717668

[B31] Li M. Zheng C. Sato T. Kawada T. Sugimachi M. Sunagawa K. (2004). Vagal nerve stimulation markedly improves long-term survival after chronic heart failure in rats. *Circulation* 109 120–124. 10.1161/01.cir.0000105721.71640.da 14662714

[B32] Loperena R. Van Beusecum J. P. Itani H. A. Engel N. Laroumanie F. Xiao L. (2018). Hypertension and increased endothelial mechanical stretch promote monocyte differentiation and activation: roles of STAT3, interleukin 6 and hydrogen peroxide. *Cardiovasc. Res.* 114 1547–1563. 10.1093/cvr/cvy112 29800237 PMC6106108

[B33] Mahadi K. M. Lall V. K. Deuchars S. A. Deuchars J. (2019). Cardiovascular autonomic effects of transcutaneous auricular nerve stimulation via the tragus in the rat involve spinal cervical sensory afferent pathways. *Brain Stimul.* 12 1151–1158. 10.1016/j.brs.2019.05.002 31129152

[B34] Majmudar M. D. Keliher E. J. Heidt T. Leuschner F. Truelove J. Sena B. F. (2013). Monocyte-directed RNAi targeting CCR2 improves infarct healing in atherosclerosis-prone mice. *Circulation* 127 2038–2046. 10.1161/circulationaha.112.000116 23616627 PMC3661714

[B35] Martelli D. Farmer D. G. McKinley M. J. Yao S. T. McAllen R. M. (2019). Anti-inflammatory reflex action of splanchnic sympathetic nerves is distributed across abdominal organs. *Am. J. Physiol. Regul. Integr. Comp. Physiol.* 316 R235–R242. 30576218 10.1152/ajpregu.00298.2018

[B36] Martelli D. Yao S. McKinley M. McAllen R. (2014). Reflex control of inflammation by sympathetic nerves, not the vagus. *J. Physiol.* 592 1677–1686. 10.1113/jphysiol.2013.268573 24421357 PMC3979618

[B37] Mazloom R. Eftekhari G. Rahimi M. Khori V. Hajizadeh S. Dehpour A. R. (2013). The role of α7 nicotinic acetylcholine receptor in modulation of heart rate dynamics in endotoxemic rats. *PLoS One* 8:e82251. 10.1371/journal.pone.0082251 24340009 PMC3858293

[B38] Mouton A. J. DeLeon-Pennell K. Y. Gonzalez O. J. R. Flynn E. R. Freeman T. C. Saucerman J. J. (2018). Mapping macrophage polarization over the myocardial infarction time continuum. *Basic Res. Cardiol.* 113:26. 10.1007/s00395-018-0686-x PMC5986831 29868933

[B39] Nuntaphum W. Pongkan W. Wongjaikam S. Thummasorn S. Tanajak P. Khamseekaew J. (2018). Vagus nerve stimulation exerts cardioprotection against myocardial ischemia/reperfusion injury predominantly through its efferent vagal fibers. *Basic Res. Cardiol.* 113:22. 10.1007/s00395-018-0683-0 29744667

[B40] Ong S.-B. Hernández-Reséndiz S. Crespo-Avilan G. E. Mukhametshina R. T. Kwek X.-Y. Cabrera-Fuentes H. A. (2018). Inflammation following acute myocardial infarction: multiple players, dynamic roles, and novel therapeutic opportunities. *Pharmacol. Ther.* 186 73–87. 10.1016/j.pharmthera.2018.01.001 29330085 PMC5981007

[B41] O’Rourke S. Dunne A. Monaghan M. G. (2019). The role of macrophages in the infarcted myocardium: orchestrators of ECM remodelling. *Front. Cardiovasc. Med.* 6:101. 10.3389/fcvm.2019.00101 31417911 PMC6685361

[B42] Peuker E. T. Filler T. J. (2002). The nerve supply of the human auricle. *Clin. Anat.* 15 35–37. 10.1002/ca.1089 11835542

[B43] Ploeger D. T. Hosper N. A. Schipper M. Koerts J. A. de Rond S. Bank R. A. (2013). Cell plasticity in wound healing: paracrine factors of M1/M2 polarized macrophages influence the phenotypical state of dermal fibroblasts. *Cell Commun. Signal.* 11:29. 10.1186/1478-811x-11-29 23601247 PMC3698164

[B44] Potts J. T. Lee S. M. Anguelov P. I. (2002). Tracing of projection neurons from the cervical dorsal horn to the medulla with the anterograde tracer biotinylated dextran amine. *Auton. Neurosci.* 98 64–69. 10.1016/s1566-0702(02)00034-6 12144043

[B45] Premchand R. K. Sharma K. Mittal S. Monteiro R. Dixit S. Libbus I. (2016). Extended follow-up of patients with heart failure receiving autonomic regulation therapy in the ANTHEM-HF study. *J. Cardiac. Fail.* 22 639–642. 10.1016/j.cardfail.2015.11.002 26576716

[B46] Rentrop K. P. Feit F. (2015). Reperfusion therapy for acute myocardial infarction: concepts and controversies from inception to acceptance. *Am. Heart J.* 170 971–980. 10.1016/j.ahj.2015.08.005 26542507

[B47] Ridker P. M. Everett B. M. Pradhan A. MacFadyen J. G. Solomon D. H. Zaharris E. (2019). Low-dose methotrexate for the prevention of atherosclerotic events. *N. Engl. J. Med.* 380 752–762. 30415610 10.1056/NEJMoa1809798 PMC6587584

[B48] Ridker P. M. Everett B. M. Thuren T. MacFadyen J. G. Chang W. H. Ballantyne C. (2017). Antiinflammatory therapy with canakinumab for atherosclerotic disease. *N. Engl. J. Med.* 377 1119–1131. 28845751 10.1056/NEJMoa1707914

[B49] Ridker P. M. MacFadyen J. G. Everett B. M. Libby P. Thuren T. Glynn R. J. (2018). Relationship of C-reactive protein reduction to cardiovascular event reduction following treatment with canakinumab: a secondary analysis from the CANTOS randomised controlled trial. *Lancet* 391 319–328. 29146124 10.1016/S0140-6736(17)32814-3

[B50] Rocha J. A. Ribeiro S. P. França C. M. Coelho O. Alves G. Lacchini S. (2016). Increase in cholinergic modulation with pyridostigmine induces anti-inflammatory cell recruitment soon after acute myocardial infarction in rats. *Am. J. Physiol. Regul. Integr. Comp. Physiol.* 310 R697–R706. 26791829 10.1152/ajpregu.00328.2015 PMC4867407

[B51] Rosas-Ballina M. Ochani M. Parrish W. R. Ochani K. Harris Y. T. Huston J. M. (2008). Splenic nerve is required for cholinergic antiinflammatory pathway control of TNF in endotoxemia. *Proc. Natl. Acad. Sci. U.S.A.* 105 11008–11013. 10.1073/pnas.0803237105 18669662 PMC2504833

[B52] Schwartz P. J. De Ferrari G. M. Sanzo A. Landolina M. Rordorf R. Raineri C. (2008). Long term vagal stimulation in patients with advanced heart failure First experience in man. *Eur. J. Heart Fail.* 10 884–891. 10.1016/j.ejheart.2008.07.016 18760668

[B53] Statz G. M. Olshansky B. (2019). Editorial commentary: vagal nerve stimulation for myocardial ischemia-reperfusion injury: hope or hype?. *Trends Cardiovasc. Med.* [Epub ahead of print]. 10.1016/j.tcm.2019.12.004 31926809

[B54] Stavrakis S. Tran N. Asad Z. Po S. (2017). Low level transcutaneous vagus nerve stimulation acutely ameliorates diastolic function in humans. *Eur. Heart J.* 38 28–38.

[B55] Tardif J.-C. Kouz S. Waters D. D. Bertrand O. F. Diaz R. Maggioni A. P. (2019). Efficacy and safety of low-dose colchicine after myocardial infarction. *N. Engl. J. Med.* 381 2497–2505. 31733140 10.1056/NEJMoa1912388

[B56] Timmers L. Pasterkamp G. de Hoog V. C. Arslan F. Appelman Y. de Kleijn D. P. (2012). The innate immune response in reperfused myocardium. *Cardiovasc. Res.* 94 276–283. 10.1093/cvr/cvs018 22266751

[B57] Todd A. J. (2010). Neuronal circuitry for pain processing in the dorsal horn. *Nat. Rev. Neurosci.* 11:823. 10.1038/nrn2947 21068766 PMC3277941

[B58] Tracey K. J. J. N. (2002). The inflammatory reflex. *Nature* 420 853–859. 10.1038/nature01321 12490958

[B59] Tran N. Asad Z. Elkholey K. Scherlag B. J. Po S. S. Stavrakis S. (2019). Autonomic neuromodulation acutely ameliorates left ventricular strain in humans. *J. Cardiovasc. Transl. Res.* 12 221–230. 10.1007/s12265-018-9853-6 30560316 PMC6579714

[B60] Trevani A. S. Andonegui G. Giordano M. Nociari M. Fontan P. Dran G. (1996). Neutrophil apoptosis induced by proteolytic enzymes. *Lab. Invest.* 74 711–721. 8600321

[B61] Uemura K. Zheng C. Li M. Kawada T. Sugimachi M. (2010). Early short-term vagal nerve stimulation attenuates cardiac remodeling after reperfused myocardial infarction. *J. Card Fail.* 16 689–699. 10.1016/j.cardfail.2010.03.001 20670848

[B62] Uitterdijk A. Yetgin T. te Lintel Hekkert M. Sneep S. Krabbendam-Peters I. van Beusekom H. M. (2015). Vagal nerve stimulation started just prior to reperfusion limits infarct size and no-reflow. *Basic Res. Cardiol.* 110:508. 10.1007/s00395-015-0508-3 PMC4549380 26306761

[B63] Vida G. Peña G. Deitch E. A. Ulloa L. (2011). α7-cholinergic receptor mediates vagal induction of splenic norepinephrine. *J. Immunol.* 186 4340–4346. 10.4049/jimmunol.1003722 21339364 PMC3083451

[B64] Vida G. Peña G. Kanashiro A. Thompson-Bonilla MdR Palange D. Deitch E. A. (2011). β2-Adrenoreceptors of regulatory lymphocytes are essential for vagal neuromodulation of the innate immune system. *FASAEB J.* 25 4476–4485. 10.1096/fj.11-191007 21840939 PMC3236627

[B65] Wang H. Yu M. Ochani M. Amella C. A. Tanovic M. Susarla S. (2003). Nicotinic acetylcholine receptor α7 subunit is an essential regulator of inflammation. *Nature* 421 384–388. 12508119 10.1038/nature01339

[B66] Wang J. Li R. Peng Z. Zhou W. Hu B. Rao X. (2019). GTS-21 reduces inflammation in acute lung injury by regulating M1 polarization and function of alveolar macrophages. *Shock* 51 389–400. 29608552 10.1097/SHK.0000000000001144

[B67] Wang Z. Yu L. Wang S. Huang B. Liao K. Saren G. (2014). Chronic intermittent low-level transcutaneous electrical stimulation of auricular branch of vagus nerve improves left ventricular remodeling in conscious dogs with healed myocardial infarction. *Circ. Heart Fail.* 7 1014–1021. 25332149 10.1161/CIRCHEARTFAILURE.114.001564

[B68] Wang Z. Zhou X. Sheng X. Yu L. Jiang H. (2015). Noninvasive vagal nerve stimulation for heart failure: was it practical or just a stunt? *Int. J. Cardiol.* 187 637–638. 25863739 10.1016/j.ijcard.2015.03.430

[B69] Warren C. M. Tona K. D. Ouwerkerk L. Van Paridon J. Poletiek F. van Steenbergen H. (2019). The neuromodulatory and hormonal effects of transcutaneous vagus nerve stimulation as evidenced by salivary alpha amylase, salivary cortisol, pupil diameter, and the P3 event-related potential. *Brain Stimul.* 12 635–642. 30591360 10.1016/j.brs.2018.12.224

[B70] Yakunina N. Kim S. S. Nam E. C. (2017). Optimization of transcutaneous vagus nerve stimulation using functional MRI. *Neuromodulation* 20 290–300. 27898202 10.1111/ner.12541

[B71] Yao W. Tai L. W. Liu Y. Hei Z. Li H. (2019). Oxidative stress and inflammation interaction in ischemia reperfusion injury: role of programmed cell death. *Oxid. Med. Cell. Longev.* 2019:6780816. 10.1155/2019/6780816 PMC6476075 31089413

[B72] Yokoyama T. Lee J.-K. Miwa K. Opthof T. Tomoyama S. Nakanishi H. (2017). Quantification of sympathetic hyperinnervation and denervation after myocardial infarction by three-dimensional assessment of the cardiac sympathetic network in cleared transparent murine hearts. *PLoS One* 12:e0182072. 10.1371/journal.pone.0182072 28753665 PMC5533449

[B73] Yu L. Huang B. Po S. S. Tan T. Wang M. Zhou L. (2017). Low-level tragus stimulation for the treatment of ischemia and reperfusion injury in patients with ST-segment elevation myocardial infarction: a proof-of-concept study. *JACC Cardiovasc. Interv.* 10 1511–1520. 28797427 10.1016/j.jcin.2017.04.036

[B74] Zamotrinsky A. Kondratiev B. de Jong J. W. (2001). Vagal neurostimulation in patients with coronary artery disease. *Auton. Neurosci.* 88 109–116. 11474540 10.1016/S1566-0702(01)00227-2

[B75] Zannad F. De Ferrari G. M. Tuinenburg A. E. Wright D. Brugada J. Butter C. (2015). Chronic vagal stimulation for the treatment of low ejection fraction heart failure: results of the NEural Cardiac TherApy foR Heart Failure (NECTAR-HF) randomized controlled trial. *Eur. Heart J.* 36 425–433. 25176942 10.1093/eurheartj/ehu345 PMC4328197

[B76] Zhang Q. Lu Y. Bian H. Guo L. Zhu H. (2017). Activation of the α7 nicotinic receptor promotes lipopolysaccharide-induced conversion of M1 microglia to M2. *Am. J. Transl. Res.* 9:971. PMC5375991 28386326

[B77] Zhang Y. Chen A. Song L. Li M. Luo Z. Zhang W. (2016). Low-level vagus nerve stimulation reverses cardiac dysfunction and subcellular calcium handling in rats with post-myocardial infarction heart failure. *Int. Heart J.* 57 350–355. 27181040 10.1536/ihj.15-516

[B78] Zhao C. Mirando A. C. Sové R. J. Medeiros T. X. Annex B. H. Popel A. S. (2019). A mechanistic integrative computational model of macrophage polarization: implications in human pathophysiology. *PLoS Comput. Biol.* 15:e1007468. 10.1371/journal.pcbi.1007468 31738746 PMC6860420

[B79] Zhou L. Filiberti A. Humphrey M. B. Fleming C. D. Scherlag B. J. Po S. S. (2019). Low-level transcutaneous vagus nerve stimulation attenuates cardiac remodelling in a rat model of heart failure with preserved ejection fraction. *Exp. Physiol.* 104 28–38. 30398289 10.1113/EP087351 PMC6312463

